# Perspectives for rare earth elements as feed additive in livestock — A review

**DOI:** 10.5713/ajas.19.0242

**Published:** 2019-08-03

**Authors:** Hujaz Tariq, Amit Sharma, Srobana Sarkar, Lamella Ojha, Ravi Prakash Pal, Veena Mani

**Affiliations:** 1 Animal Nutrition Division, ICAR-National Dairy Research Institute (Deemed University), Karnal, Haryana 132001, India; 2 Department of Animal Nutrition, Guru Angad Dev Veterinary and Animal Sciences University, Ludhiana, Punjab, 141004, India; 3 ICAR- Central Sheep and Wool Research Institute, Avikanagar, Rajasthan, 304501, India

**Keywords:** Antimicrobial Resistance, Livestock Production, Rare Earth Elements

## Abstract

There is a need for newer feed additives due to legal prohibition on inclusion of growth promoting antibiotics in livestock diets in several countries due to antimicrobial resistance. In this context, rare earth elements (REE) have gained attention among animal nutritionists as potential growth promoters. Currently, several studies have reported better weight gain, milk production, egg laying capacity and feed conversion efficiency among different breeds of farm animals following supplementation with REE, with however largely inconsistent results. Furthermore, REE supplementation has also shown to improve ruminal fibrolytic and proteolytic activities as well as flavor of meat with negligible residues in edible tissue, however the mechanism behind this action is still unclear. According to existing research, due to their poor absorption and similarity with calcium REE might exert their action locally on gut microbial populations within the gastrointestinal tract (GIT). Moreover, REE have also shown anti-inflammatory, anti-oxidative as well as immune stimulating effects. The present review aims to broaden the knowledge about use of REE as feed additives for livestock and sum up efficacy of REE supplementation on performance and health of animals by comparing the findings. Till date, researches with REE have shown properties that make them a promising, new and safe alternative feed additive but further exploration is recommended to optimize effects and clarify discrepancy of various results before practical proposals can be drafted.

## INTRODUCTION

Feed additives are ingredients/chemical compounds or combinations thereof which are added to the basal ration to fulfill specific requirement or to improve weight gain, feed efficiency and control diseases in farm animals. Feed additives can be broadly classified into nutrient (amino acids, minerals, and vitamins) and non nutrient (antibiotics, hormones, enzymes, prebiotics, yeast culture, pellet binder, antioxidants etc.) feed additive [1]. Antimicrobial compounds such as antibiotics were previously used in feeds for therapeutic purposes to cure and prevent infectious diseases. But soon the growth promoting effect of antibiotics came into limelight under intensive animal rearing system; thereafter these were added at sub therapeutic doses to animal feeds as an additive for nearly 6 decades. These compounds also improved feed utilization (2% to 5%) and reduced morbidity as well as mortality due to clinical and subclinical diseases [2,3]. However, extensive use of antibiotics at therapeutic or subtherapeutic doses over long period of time provides favorable conditions for proliferation of antimicrobial resistant microorganisms in animals, plants and soil [1].

At present global concern regarding antimicrobial resistance and transference of resis tant strains gene from animal to human is rising [4–6]. Therefore, the potential risks associated with development of antibiotic resistant bacteria and its transmission led to the ban on use of feed antibiotics as growth promoters in European Union since 2006. This ban endorsed scientific community and animal feed industry to actively explore alternatives for feed antibiotics which could improve feed efficiency, weight gain and manipulate rumen fermentation [7]. As a result, the rare earth elements (REE) have gained interest among animal nutrition research for its efficacy as a feed additive in substitution to antibiotics.

The REE are not a newer feed additive as these have been successfully used as fertilisers in plant production and as growth promoter in animal production for many decades in China [8]. Till date numerous scientific reports indicated that a small amount of REE mixtures in the diet of farm animals increased live weight gain, feed efficiency and milk/egg production [9,10]. The REE are naturally occurs in the environment and consist of three members of group IIIB, namely scandium (Sc; atomic number 21), yttrium (Y; atomic number 39), lanthanum (La; atomic number 57) and 14 chemical elements from group IIIA of the periodic table called lanthanoids (atomic numbers 58–71) *viz*. cerium (Ce), praseodymium (Pr), neodymium (Nd), promethium (Pm), samarium (Sm), europium (Eu), gadolinium (Gd), terbium (Tb), dysprosium (Dy), holmium (Ho), erbium (Er), thulium (Tm), ytterbium (Yb), lutetium (Lu), and the elements Y and Sc. These are frequently divided into two subgroups: the light or Ce group and the heavy or Y group. The Ce group consists of the elements La, Ce, Pr, Nd, Pm, Sm, and Eu, whereas Y group comprises the elements Gd, Tb, Dy, Ho, Er, Tm, Yb, Lu, and Y [11]. The importance of various inorganic elements (commonly known as minerals) in human, animal and plant nutrition are very familiar and are required for various metabolic functions [12–14]. Now, among inorganic elements, REE emerges as new generation growth promoter feed additive alternative to feed antibiotics for livestock. The aim of present review is to broaden the knowledge about use of REE as feed additive in livestock, its effect on health and performance of animals as well as its accumulation in animal tissue.

## HISTORICAL ASPECTS RELATED TO RARE EARTH ELEMENTS

The history of the discovery of REE is very old and first element to be discovered was in 1788, by the Swedish army lieutenant Karl Axel Arrhenius, who collected the black mineral ytterbite (named after the nearby Swedish town). After that, Gadolin in 1794, isolated ytterbia from the mineral ytterbite using fractional crystallization and explained their chemical properties. This created the interest among many scientists and with development of various technology like spectroscopic methods of analysis and the exploration of electrochemical separation led to discovery of various REE. The latest REE discovered was Pm in 1947 through the use of ion exchange chromatography from fission fragments of uranium.

## OCCURRENCE, MINERAL SOURCES, AND SEPARATION TECHNIQUE

The term “rare earths” is a misnomer as these elements are neither rare nor earths. The word “rare”, relates to the considerable difficulties in separating one REE from another because of their close similarity in physical and chemical properties. All REE are more common than Ag or Hg [11] and Tm, the rarest of the REE, are found more often than gold, platinum or iodine [15]. The term “earths” was used in the 18th century for describing alkaline metal oxides, as they were first identified as rare earth oxides. Rare earth elements are widely distributed and mainly occur as phosphates because they have strong affinity for phosphate. Moreover, oxides, silicates, carbonate and halogen compounds of REE are also present in mineral deposits under natural conditions [16]. There are more than two hundred mineral deposits known to contain REE and among which few of them are useful for industrial production.

Several methods of separating rare earths have been iden tified, but none are considered to be universal. Usually there are choices between either acidic (using sulphuric acid) or alkaline (using caustic soda) methods of breakdown, which are temperature dependent. High purity isolates for commercial purposes are mainly obtained by solvent extraction while ion exchange resins are used in special cases [15].

## MECHANISM OF ACTION AND BIOLOGICAL EFFECTS OF RARE EARTH ELEMENTS

**Figure 1:**
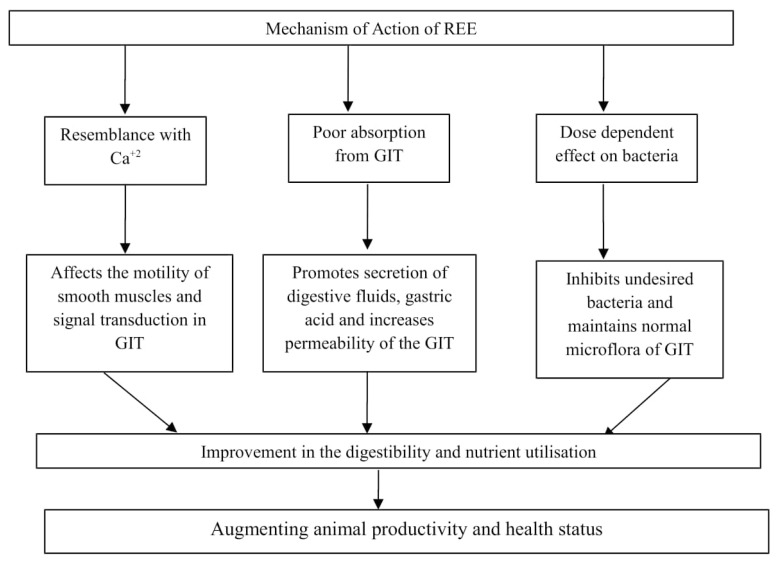
Proposed mechanism of action of rare earth element. REE, rare earth elements; GIT, gastrointestinal tract.

The scientific literature and evidences on the mechanism of REE which enhances the performance of animals are limited and not well established. However, various biological effects based on results of different feeding trials on supplementation of REE in the diet of animals have been proposed (Figure 1) viz. antibacterial property, antioxidant nature, increased hormonal, and enzyme activities, proliferation of specific cells, stimulation of immune system, enhancement of digestibility and nutrient utilization [10,17]. These effects of REE can be either due to their similarity with calcium (radius of Ca is very close to lanthanoids) or poor absorption from the gastrointestinal tract (GIT) [18,19]. Moreover, it is believed that the little amount of absorbed REE affected various physiological process like hormonal concentration, enzyme activity, activation of immune system, metabolism of nutrients (protein or lipids) and cell proliferation [20,21]. Further, REE are supposed to have hormetic effect on microbes i.e. concentration-related effects, by improvements in biological events at low levels, followed by inhibitory effects at increasing concentrations [22–24].

### Interaction with intermediate metabolism and antibacterial properties of rare earth elements

Muroma [25], first observed the antibacterial effect of REE and reported its dose dependent activity i.e. at higher concentrations (10^−4^ to 10^−2^ mol/L) these elements inhibit bacterial growth mainly Gram negative, whereas at lower concentrations (10^−5^ mol/L) bacterial growth was stimulated. Similarly, Zhang et al [26] concluded that the growth of *Escherichia coli* (*E. coli*), *Bacillus pyocyaneus*, *Staphyloccous aureus*, *Leuconostoc*, and *Streptococcus faecalis* were inhibited by Ce (10^−3^ mol/L to 10^−2^ mol/L). Zhao et al [27] also reported that Ce ions at concentrations below 350 μg/mL had a stimulatory effect on the growth of *E. coli*, whereas concentrations at or above 400 μg/mL had an inhibitory effect. Stimulatory effect of REE was explained by Talburt and Johnson [28] as they reported that oxalic acid produced by *Aspergillus niger* combines with REE to form insoluble oxalates and resulting in detoxification mechanism permitting further growth of this microbes. More recently, Liu et al [29] explained inhibitory of REE using La nitrate effect on the growth of *E. coli* B. They observed that there was an abnormal “eruption of heat” phenomenon at high concentration (500 mg/L) led to damage the outer cell membrane and increases its permeability along with reduced proton-electron potential energy across the cell membrane. Further, the growth of the cells was inhibited due to scarceness of energy adenosine triphosphate (ATP) as energy could not be translated into ATP effectively in the course of oxidative phosphorylation and resulted in more heat release. Therefore, from above studies it can be considered that REE has hormetic properties so use of REE in feed might enhance animal performance by influencing the development of desired bacterial species within the GIT selectively in a dose dependent manner.

### Improvement of digestibility and nutrient utilization

In case of rats absorption of REE from GIT was reported to be less than 0.01% [30], while, absorption factors for chickens ranged from 10^−3^ to 10^−4^ [31]. Hence, orally supplemented REE generally accumulates within the GIT and promotes the secretion of digestive fluids and influences the permeability of intestine, thereby enhancing the absorption of nutrients as well as digestibility [32–34]. Further, these elements show resemblance with Ca, not only in size and bonding but also in coordination geometry and donor atom preference. So, these elements may affect the Ca^2+^-dependent release of chemical mediators [18]. Further, REE interact with biomolecules by binding to Ca^2+^ or Mg^2+^ binding sites of calmodulin, ATPase of sarcoplasmic reticulum, cystatin and phosphatidylserine. The binding mode of various REE to calmodulin is varied a lot as Lu^3+^ and Er^3+^ bind like Ca^2+^, Eu^3+^, and Tb^3+^ binding the opposite order from Ca^2+^, whereas, La^3+^ and Nd^3+^ binding a mode between them [18]. On the other hand, the poor absorption of REE is supposed to be associated with its accumulation in GIT which further affects the growth of harmful bacteria resulting in anti-bacterial, anti-inflammatory effects as well as increased digestibility of nutrients in the GIT. These mechanisms show that REE can stimulate the digestive micro-organisms or enzymes and increase ruminal fibrolytic well and proteolytic activities. Moreover, the palatability of diet remains unaffected as these elements are generally tasteless in nature. Hence, beneficial properties of REE supplementation in the diet of animals may be through its effect on feed intake, passage rate and nutrient digestibility [30,10]. The pathways through which these absorbed REE acts on physiological processes remains indistinct, so the essentiality and physiological roles of REE in animals and human beings are inconclusive.

## ROLE OF RARE EARTH ELEMENTS IN ANIMAL HUSBANDRY

Supplementation of animal feeds with REE has been practiced in China for many decades. After the ban on antibiotic growth promoters, these elements have come into focus as newer natural feed additives in substitution to antimicrobials. Numerous studies reported that certain dose of REE in the diet of animals could improve their health, body weight (BW) gain, feed conversion efficiency, milk and egg production [9,35].

### Non ruminants

#### Pig

In case of pig promising growth promoting effects of REE were observed. It was reported that supplementation of a mixture of REE (38% LaCl_3_·6H_2_O, 52.1% CeCl_3_·6H_2_O, 3% PrCl_3_·6H_2_O, and 6.9% chlorides of other REE) at a dose of 300 mg/kg of feed resulted in higher BW gain (19% and 12%) and feed conversion ratio (FCR) (11% and 3%) in growing and fattening pigs respectively. Moreover, it was observed that supplementation of REE for 3 months had no residual effect in the muscle, liver and kidneys of pigs [21]. In this context, apart from REE mixture individual supplementation of La at a dose of 100 mg/kg dry matter (DM) in diet increased average daily gain (ADG) and FCR by 12.95% and 6.78% respectively [9]. Further, in the above study it was also reported that La residues in selected organs (longissimus muscle, liver, kidney, and pancreas) remained unaltered with supplementation of dietary La. The non-residual effect of REEs in tissues may be due to poor absorption or rapid elimination of REEs from the body of animals. Hence, health of the animals and safety of animal products are not influenced by supplementation of REE mixture or La in the diet of pigs. Recently, organic form of REE i.e. REE-enriched yeast (RY; containing 2.82% La, 4.71% Ce, 40.3% distillers dried grains with solubles (DDGS) and 52.17% yeast) was studied for its effect on feed intake and animal performance in comparison to supplementation of tiamulin antibiotic in the diet of finishing pigs [36]. Animals in control group were fed on only basal diet, whereas in RY groups, they were supplemented with graded levels i.e. 500, 1,000, and 1,500 ppm RY and in antibiotic group tiamulin 500 mg/kg was added in the feed along with basal diet. It was observed that ADG, average daily feed intake, gain to feed ratio, apparent total tract digestibility of DM and gross energy were enhanced linearly with graded levels of RY as compared to control group only. The performance of pig supplemented with RY remained at par to pigs supplemented with antibiotics tiamulin. Therefore, REE could be considered as a possible effective and safe substitute to feed antibiotics. The improved performance of pigs supplemented with REE may be related to its antibacterial properties. Although, Kraatz et al [37] observed that addition of REE-citrate (a premix containing predominantly organic citrate compounds of 23.3% La, 68.2% Ce, 8.0% Pr, and 0.4% Nd) at 200 mg/kg DM in the diet of weaning piglets had no significant effect on FCR and fecal microbiota. Similarly, Halle et al [38] also reported non-significant effect of REE supplementation on nutrient digestibility in pigs. The variations in the results of different studies may be endorsed to the dose of REEs along with the type used for supplementation in different trials. Various other studies regarding the effect of REE on swine production and health have been highlighted in Table 1.

#### Poultry

Effect of REE supplementation in poultry diets have been summarized in Table 2. Addition of REE to layer diets (30 to 1,000 mg/kg feed) resulted in increased egg production (3% to 15%), hatchability (5% to 15%) and improved feed conversion efficiency (2% to 22%) as well as BW gain (4% to 14%) [39,40]. Recently, Cai et al [41] observed that supplementation of RY at 1,500 mg/kg (containing 42.3 mg/kg La, and 70.65 mg/kg Ce) during starter and growing period resulted in improved nutrient digestibility and meat quality. However, there was no significant influence on growth performance, relative organ weight and excreta microflora. Earlier He et al [42] compared the effect of dose and form of REE compounds i.e. organic and inorganic REE supplementation on broiler performance. They supplemented REE-chloride (containing LaCl_3_ 380 mg/kg; CeCl_3_ 520 mg/kg; PrCl_3_ 30 mg/kg, and chlorides of other REE 70 mg/kg) at 40 mg/kg and REE-citrate (La-citrate, 210 g/kg; Ce-citrate 670 g/kg; Pr-citrate, 120 g/kg) at 70 mg/kg in the diet of broiler. It was observed that dietary REE-citrate improved BW gain by 5.0% while supplementation of REE-chloride showed no improvement as compared to control. In another experiment they supplemented REE-chloride at 70 mg/kg and REE-citrate at 70 and 100 mg/kg in the broiler diet. Results showed that only FCR was improved by 3.4% in group supplemented with REE-citrate at 70 and 100 mg/kg as compared to RRE chloride and control groups. However, blood serum biochemical parameters were not significantly affected by REE in the diets. From above results it can be assumed that dietary supplementation of REE-citrates at 70 mg/kg had beneficial effect on growth performance of broilers without affecting carcass composition and health of the broilers. In contrast to above studies, Igbasan and Adebayo [43] observed that supplementation of La either as La chloride (LaCl_3_) or La oxide (La_2_O_3_) at 100, 200, 300, or 400 mg/kg DM in the diet of broiler did not influence growth, carcass quality, haematological and biochemical parameters.

#### Ruminants

Studies regarding the effect of REE on production performance of ruminants have been summarized in Table 3. REE supplementation in ruminants had variable effect on ADG, feed intake, rumen fermentation and milk production. Literature summarized by Redling [10] reported that addition of rare earth oxides (mainly La 22%, Ce 45%, and Nd 15% oxides) and rare earth nitrates consisting of (38%) rare earth oxides (22% La_2_O_3_, 45% Ce_2_O_3_, 15% Nd_2_O_3_) at 600 and 800 ppm in the diet of fattening cattle and dairy cows improved daily gain and milk production, respectively. Furthermore, Liu et al [33] observed that supplementation of LaCl_3_ at 900 mg per steer per day significantly (p<0.05) improved rumen fermentation and feed digestion. Studies revealed that REE supplementation decreased the ruminal pH, which resulted in increased population of cellullytic bacteria, which in turn enhance the digestibility of feed in ruminant. Although, organic form i.e. REE- citrate (REE 25.3% which contains 57.9% Ce, 34.0% La, 6.5% Pr, 1.6% of other REE) at 200 mg/kg DM in pre-ruminants did not affect feed intake and performance parameters [8]. Similar, reports were reported by Renner et al [17] when they supplemented REE-citrate (containing 34.30% La, 58.09% Ce, and 7.61% other REE) up to 300 ppm in growing bulls, however, it was observed that the proliferation of peripheral blood mononuclear cells was significantly higher in groups supplemented with REE-citrate at 300 ppm, thus REE may play potential role in boosting bovine immune system.

## SAFETY ASPECTS OF RARE EARTH ELEMENTS SUPPLEMENTATION TO ANIMALS AND ITS ACCUMULATION IN ANIMAL PRODUCTS

Results of various studies reported that concentrations of REE in liver and muscle is weakly affected by the dose of REE supplementation in diets of poultry, pig and fattening bull [21,42,19]. Moreover, RE are not highly toxic as LD_50_ values for IV-injected REE are 10 to 100 mg/kg/BW and those of IP-injected REE are 150 to 700 mg/kg/BW. Toxicity of REE through oral route is very low as only very small amounts of REE are absorbed in the GIT [44]. When rats were given higher levels of REE (LaCl_3_·7H_2_O at 1,000 mg/kg BW/d for 28 days) orally, it induced hepatotoxic effect and caused irritation to the stomach mucosa [24]. Hence, it can be supposed that oral supplementation of REE in the diet of animals may pose similar health risk like that of table salt. Further, either due to poor absorption or rapid elimination of REEs from the body of animals the health risks to humans consuming edible tissues from these animals can be regarded as negligible. However, due to limited scientific information there is controversy regarding health benefits and toxic effects of REE on human health [23].

## CONCLUSION AND POSSIBLE FUTURE AREAS OF RESEARCH

After reviewing the complied and cited literature, it is difficult to clarify the biological role of REE on animal performance as there is huge discrepancy among the results of various trials conducted on pig, poultry and ruminants. In case of growing pigs supplementation of REE-chloride mixture up to 1,500 ppm in the diet improved BW gain and FCR by 19% and 12%, respectively along with higher count of fecal *Lactobacillus*. However, REE-citrate mixture supplementation up to 800 ppm had no effect on pig performance at all. In case of poultry, REE- citrate mixture supplementation up to 100 ppm in diet of broiler birds improved weight gain and FCR by 5% and 3.4%, while the supplementation of RRE-chloride mixture showed no effect. Supplementation of REE-chlorides, nitrates, oxide up to 900 ppm and REE-citrate up to 300 ppm in the diet of cattle and sheep respectively, had positive impact on animal performance by improving rumen fermentation and nutrient digestibility. But, REE-citrate supplementation up to 300 ppm had no impact on rumen fermentation and performance of cattle. Apart from above findings, there are few studies which reported that REE had similar impact on fecal micro-biota and growth performance in animals when compared antibiotics supplementation. Moreover, it was also observed that the concentration of REE in various edible tissues was not affected by dose of REE in the diet. Hence, REE might be considered as an alternative to antimicrobial compounds as these are capable of augmenting performance in both ruminants and non-ruminants without any residual effect on edible tissue. Although, there is a need of further research to elucidate the pathways through which REE exerts its action on various physiological processes.

## Figures and Tables

**Table 1:** Literature summary of effect of REE supplementation in pigs

Dose	Results and conclusions	Reference
100 mg/kg DM La for 30 days	Significant increase in ADG and feed intake (13.3% and 5.4%, respectively) Significantly improved in feed conversion ratio (8.5%) Significant increase in levels of serum thyroid hormones (T_3_ and T_4_)	Xu et al [45]
Cerium (Ce) at 0.5 to 10 μmol/L and >10 μmol/L	Enhanced the activity of α-amylase from porcine pancreas at lower concentration (0.5 to 10 μmol/L) Inhibited the activity of α-amylase at higher concentration of Ce^3+^ (>10 μmol/L)	Wang et al [46]
REE at 100, 200, 400, and 800 mg/kg of DM as citrate-bound REE (having lanthanum 30, cerium 55, praseodymium 5, and neodymium 10%)	REE supplementation did not disturb the health of animals Low concentrations (100 mg/kg DM) of REE increased weight gain Higher concentrations (200 mg/kg DM) of REE adversely affected animal growth performance	Förster et al [47]
0.1% peptide-bound REE mineral-yeast (mixture contained 35.3 lanthanum, 25.2 cerium, 10.2 praseodymium, and 29.3 of several minerals which were present in trace amounts)	Significantly increased in total tract digestibility of DM, CP, and GE	Han and Thacker [48]

REE, rare earth elements; DM, dry matter; ADG, average daily gain; CP, crude protein; GE, gross energy.

**Table 2:** Literature summary of effect of REE supplementation in poultry

Dose	Results and conclusions	Reference
250 mg/kg (La 100 mg, Ce 150 mg) and 500 mg/kg (La 200, Ce 300 mg) of REE in the diet of laying hen	Significant increase in plasma Ca and P levels at first and second month when supplemented group treated with REE at 250 mg/kg Whereas in group supplemented with 500 mg/kg REE plasma Ca and P had significantly increased only during first month of the trial No significant changes were reported in second month of the trial Non-significant effect on total protein, albumin and globulin level	Reka et al [49]
Lanthanum oxide (La_2_O_3_; having 85.3% La) at 100 (85.3 ppm La), 200 (171 ppm La) and 300 (256 ppm La) ppm to starter and finisher diet of broiler	Improvement in the total weight gain over the control on supplementation of La at 171 ppm Relatively lower counts of bacteria were obtained in group supplemented with 85.3 ppm La	Agbede et al [50]
Lanthanum oxide at 0, 100, 200, 300, or 400 mg/kg) in the diet of laying hen	Significant increase in Haugh unit and eggshell breaking strength Significantly decreased thiobarbituric acid reactive substance (TBARS) values in egg yolk Non-significant effect on SOD and GPx values Significant decrease in serum MDA concentration Non-significant difference in serum Ca and P level	Durmus and Bolukbası [51]
Cerium oxide at 0, 100, 200, 300, or 400 mg/kg in the diet of laying hen	Non-significant effect on feed intake and egg weight Egg production and feed conversion rate were improved by maximum level of cerium oxide (at 400 ppm) Significant decrease in SOD and MDA concentration Significant increase in serum Ca and P concentration	Bolukbası et al [52]

REE, rare earth elements; SOD, superoxide dismutase; GPx, glutathione peroxidase; MDA, malondialdehyde.

**Table 3:** Literature summary of effect of REE supplementation in ruminants

Dose	Results and conclusions	Reference
REE-citrate at 100, 200, and 300 mg/kg DM (having Ce 57.9%, La 34.0%, and Pr 6.5%) in the diet of fattening bulls.	Non-significant effect in feed-to-gain ratio, ME-to-gain ratio and digestibility of nutrients due supplementation of REE	Schwabe et al [7]
REE-citrate at 100, 200, and 300 mg/kg DM (having Ce 57.9%, La 34.0%, and Pr 6.5%) in the diet of fattening bulls.	Significantly linear increase the concentrations of REE (lanthanum [La], cerium [Ce], and praseodymium [Pr]) in the liver, kidneys and rib bone While, the concentration in muscle was not influenced Risk to humans from consuming of edible tissue of REE supplemented animals can be regarded as negligible	Schwabe et al [19]
REE-citrate (Ce 56.8%, La 35.0%, and Pr 6.5%) at 100, 200, and 300 mg/kg DM in diet of sheep	Significantly decrease in ruminal pH Quadratically decreased in ruminal ammonia content with increasing REE supplementation Other ruminal parameters like total volatile fatty acids concentration and acetate to propionate ratio were also affected by REE supplementation Negative effect on growth of several rumen bacteria Digestibility of various nutrients and urinary excretion of purine derivatives were also increased with increasing REE addition	Xun et al [53]
LaCl_3_, CeCl_3_, or PrCl_3_ at 204 mg/kg DM to the basal ration of beef cattle	Linear increase in NDF digestibility and reduced enteric CH_4_ emissions Significant decreased in total N excretion and urinary N excretion, increased N retention Total urinary PD were linearly increase Non-significant effect in N retention, urinary PD, microbial N flow and plasma biochemical parameters	Lin et al [54]
Cerium chloride (CeCl_3_) at 0, 80, 160, and 240 mg/kg DM in beef cattle	Significant increase in NDF digestibility and N retention Significantly decreased the molar ratio of rumen acetate to propionate, total N excretion, urinary N excretion and CH_4_/kg DMI	Lin et al [55]
Lanthanum oxide at 100, 200, and 300 mg/kg in the diet of sheep	Significant improvement in daily weight gain and total weight gain Non-significant effect on AST, ALT, cholesterol, urea, total protein, albumin, and globulin	Adu et al [56]
*In-vitro* addition of REE chloride mixture (LaCl_3_ 380 mg/kg; CeCl_3_ 521 mg/kg, PrCl_3_ 30 mg/kg and chlorides of other REE 69 mg/kg) at 400 and 800 mg/kg	No effect on ruminal VFA concentration Impact on VFA profile was marginal Linear increase in ruminal true digestibilities of organic acid, acid detergent fibre and crude protein with increasing REE supplementation	Yang et al [34]

REE, rare earth elements; ME, metabolizable energy; NDF, neutral detergent fiber; PD, purine derivatives; DMI, day matter intake; AST, aspartate amino transferase; ALT, alanine amino transverse; VFA, volatile fatty acid.
